# Phosphopeptide interactions of the Nbs1 N-terminal FHA-BRCT1/2 domains

**DOI:** 10.1038/s41598-021-88400-7

**Published:** 2021-04-27

**Authors:** Kyungmin Kim, Thomas W. Kirby, Lalith Perera, Robert E. London

**Affiliations:** Genome Integrity and Structural Biology Laboratory, National Institute of Environmental Health Sciences, NIH, Research Triangle Park, NC 27709 USA

**Keywords:** Structural biology, Molecular modelling, Biochemical assays

## Abstract

Human Nbs1, a component of the MRN complex involved in DNA double strand break repair, contains a concatenated N-terminal FHA-BRCT1/2 sequence that supports interaction with multiple phosphopeptide binding partners. MDC1 binding localizes Nbs1 to the damage site, while binding of CDK-phosphorylated CtIP activates additional ATM-dependent CtIP phosphorylation, modulating substrate-dependent resection. We have investigated the phosphopeptide binding characteristics of Nbs1 BRCT1/2 based on a molecular modeling approach that revealed structural homology with the tandem TopBP1 BRCT7/8 domains. Relevance of the model was substantiated by the ability of TopBP1-binding FANCJ phosphopeptide to interact with hsNbsBRCT1/2, albeit with lower affinity. The modeled BRCT1/2 is characterized by low pSer/pThr selectivity, preference for a cationic residue at the + 2 position, and an inter-domain binding cleft selective for hydrophobic residues at the + 3/ + 4 positions. These features provide insight into the basis for interaction of SDT motifs with the BRCT1/2 domains and allowed identification of CtIP pSer347- and pThr847-containing phosphopeptides as high and lower affinity ligands, respectively. Among other binding partners considered, rodent XRCC1 contains an SDT sequence in the second linker consistent with high-affinity Nbs1 binding, while human XRCC1 lacks this motif, but contains other phosphorylated sequences that exhibit low-affinity binding.

## Introduction

The Mre11-Rad50-Nbs1 (MRN) complex plays a central role in the processing of DNA double-strand breaks (DSBs) by directing initial end processing activities that largely determine selection of the repair pathway^1,2^. It also initiates an intricate set of intracellular events that involve kinase signaling cascades, cell-cycle checkpoint activation, and recruitment of additional DNA repair proteins^3–5^. Most of the DNA-damage-checkpoint signaling functions of this complex are supported by Nbs1 through its interactions with the mediator of DNA damage checkpoint 1 (MDC1), C-terminal binding protein-interacting protein (CtIP), ATM serine/threonine kinase, and other proteins^3,6–14^. The mammalian Nbs1 N-terminal FHA-BRCT1/2 domains form bipartite complexes with polyphosphorylated MDC1 and CtIP, that involve both the FHA and BRCT domains^7,15–17^. Mutations in these domains result in hypersensitivity to ionizing radiation and phenotypic characteristics of Nijmegen breakage syndrome (NBS)^18^.

Although substantial structural, mutational, and binding data are available for the *S. pombe* Nbs1 and Ctp1 orthologs^3,9,19,20^, there are sufficient differences with the human proteins to limit direct extrapolation of these results. In particular, the yeast Nbs1 BRCT1/2 domains do not interact directly with phosphopeptides. Human MDC1 contains a highly phosphorylated segment, previously referred to as the MDC1-SDT sequence, residues141-621^15,17,21^, that includes multiple CK2 phosphorylated pSer-Asp-pThr repeats. Interaction of these motifs with both the N-terminal FHA and BRCT1/2 domains of human Nbs1, leads to retention of Nbs1 at the DNA damage-modified chromatin^22^. Alternatively, interaction of the Nbs1 N-terminal domains with CDK-phosphorylated CtIP motifs leads to additional ATM-dependent CtIP phosphorylation in a substrate-specific manner^7^. The phosphorylated CtIP-Nbs1 complex activates the MRE11-RAD50 nuclease resecting the DNA break. Targeted disruption of Nbs1 in mice leads to defects that are nearly identical to those observed in ATM^−/−^ mice^23^. Insight into the nature of the CtIP-Nbs1 interaction is also of interest because interference with ATM function is under evaluation as an anti-cancer strategy^24^.

In addition to the interactions with MDC1 and CtIP, there is evidence that the Nbs1 N-terminal domain may also recognize CK2-phosphorylated motifs in XRCC1^8,25^, perhaps as a means of recruiting its Lig3α binding partner. The XRCC1 pS518-pT519 motif has been shown to support interactions with the FHA domains present in three different proteins: polynucleotide kinase/phosphorylase (PNKP); aprataxin (APTX); and aprataxin and polynucleotide kinase like factor (APLF)^26–28^. The very unusual ability of Nbs1 to recognize both CK2- and CDK-phosphorylated motifs as well as the possible additional interactions with phosphorylated XRCC1 motifs motivated the studies reported here.

## Results

### Phosphopeptide recognition by the FHA domain

**Figure 1:**
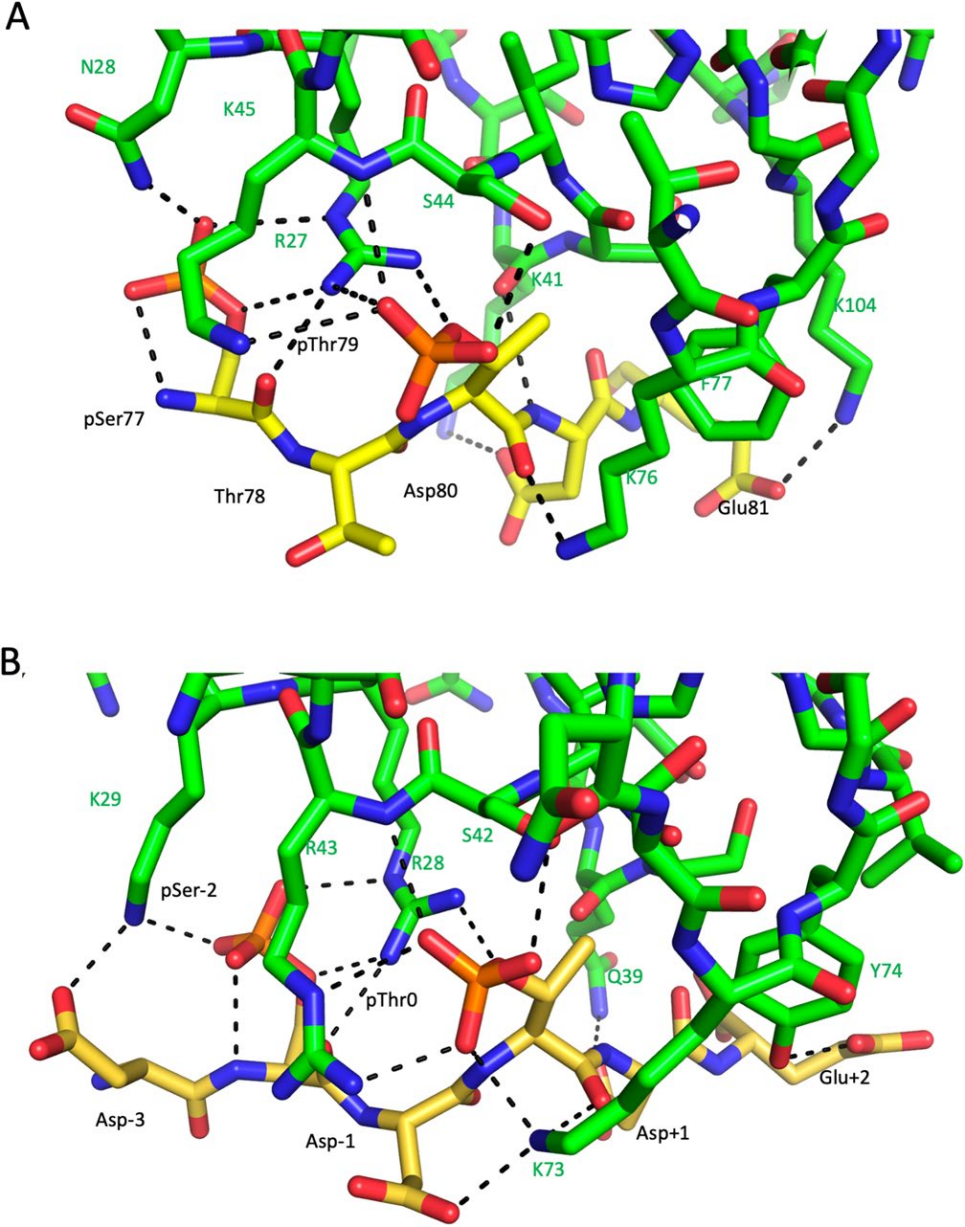
Phosphopeptide interactions with the Nbs1 FHA domain. (**A**) Peptide-protein interface of the previously reported complex of the *S. pombe* Nbs1 FHA domain with a phosphorylated Ctp1 peptide (PDB: 3HUF^9^). The disordered sidechain of the Ctp1 N-terminal pSer^77^ residue was modeled into the structure using PyMol (www.pymol.org). The pSer phosphate oxygens form H-bonds with Arg27, Asn28, and with the pSer backbone NH. (**B**) The peptide binding interface of the modeled hsNbs1 FHA domain. The initial peptide placement was obtained by overlaying the modeled structure with 3HUF and mutating the bound Ctp1 peptide to a generic SDT sequence present in MDC1. The pSer bridging oxygen is H-bonded to Arg28 and the non-bonding phosphate oxygens form H-bonds with Arg28, Lys29, and the pSer NH. The peptide pThr bridging oxygen is also H-bonded with Arg28, and the non-bridging oxygens form H-bonds with Ser42, Arg43 guanidino and backbone NH, and with the Lys73 amino sidechain. The model also shows extensive H-bonds with the peptide backbone (indicated by dashed black lines). Stick models of the Nbs1 FHA domains are shown in green, and the bound peptides in yellow. H-bond distances are summarized in Table S1.

Figure 1A shows the peptide-FHA domain interface of the previously reported Ctp1-*S. pombe* Nbs1 N-terminal domain crystal structure (PDB: 3HUF^9^). In making this figure, the disordered pSer sidechain was reintroduced using PyMol (www.pymol.org). The central binding motif involves formation of H-bonds between Arg27 and the phosphate groups of both pSer and pThr, creating a compact recognition region which differs from more frequently encountered peptide-FHA domain interfaces that involve a more extended recognition surface. The corresponding human Nbs1 FHA domain was modeled from structure 3HUF using the Swiss Model server^29^. The sequences and domain boundaries of the three hsNbs1 N-terminal domains are given in Figure S1. The structure was then overlaid with the *S. pombe* complex to obtain an initial position of the bound peptide. For greater relevance, the Ctp1 peptide was mutated to a generic Asp-pSer-Asp-pThr-Asp-Glu sequence more typical of MDC1 (Fig. 1B). In addition to supporting CK2-dependent phosphorylation and direct binding interactions, electrostatic repulsion among the anionic residues may favor an extended conformation similar to the bound form. The modeled hsFHA-MDC1 peptide complex supports the conclusion that the mode of peptide binding is well conserved. Replacement of Lys45 in spNbs1 with Arg43 in hsNbs1 supports enhanced interactions with the pThr phosphate oxygens and replacement Asn28 with Lys29 also introduces an additional salt bridge with pSer. H-bond distances are summarized in Table S1.

### TopBP1 BRCT7/8 as a model for Nbs1 BRCT1/2 and predicted phosphopeptide binding characteristics

**Figure 2:**
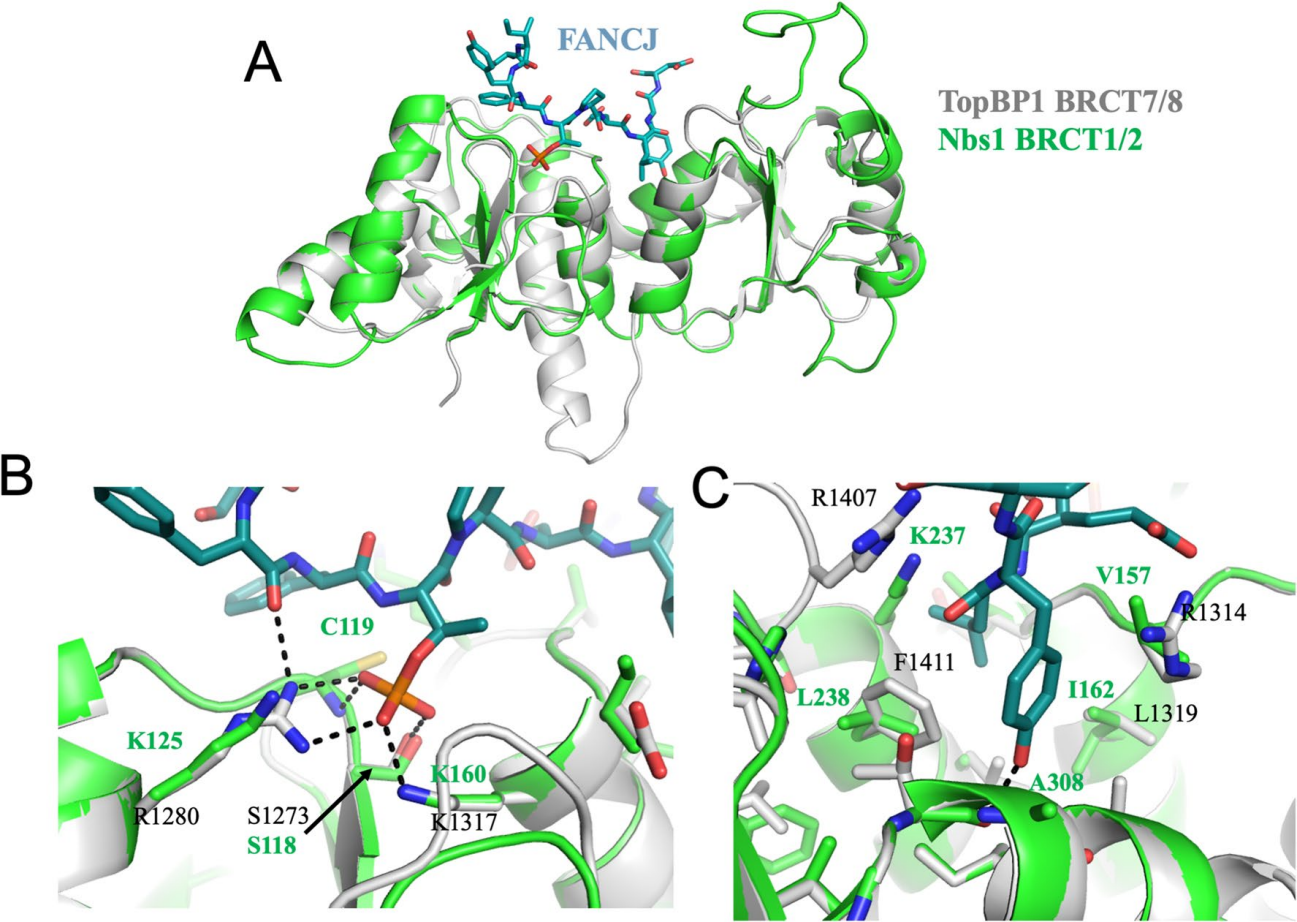
Comparison of TopBP1 BRCT7/8 domains with Modeled Nbs1 BRCT1/2 domains. (**A**) Overlaid ribbon diagrams of TopBP1 BRCT7/8 (gray) in complex with a bound FANCJ phosphopeptide (teal stick model), PDB: 3AL3^31^ with modeled hsNbs1 BRCT1/2 domains (green). The lower panels show expanded stick representations of selected residues in TopBP1 and modeled hsNbs1 BRCT1/2 domains corresponding to: (**B**) the pThr binding region, and (**C**) the hydrophobic interdomain binding pocket. The pThr binding residues Ser118, the residue 119 backbone, and Lys160 are conserved, while Arg1280 is replaced by Lys125 in Nbs1, increasing the relative pSer/pThr binding affinity. Dotted lines show pThr-TopBP1 H-bond interactions in structure 3AL3.

Although crystal structures of the *S. pombe* Nbs1 FHA-BRCT1/2 domains provide a useful basis for understanding the behavior of the human Nbs1 FHA domain^3,9^, they provide less insight into the nature of the tandem BRCT1/2 phosphopeptide binding site. Attempts to gain further insight into the structure and behavior of these domains by using the Swiss Model server^29^ identified hsTopBP1 BRCT7/8 domains as a template for Nbs1 BRCT1/2 despite a low 18.4% sequence identity. The modeled structure (Fig. 2) provides a consistent identification of some of the residues previously suggested to mediate phosphopeptide binding^3^. The structure includes an ~ 18 residue disordered loop in the BRCT2 domain, ~ Gly274–Gln291, that corresponds to a disordered segment, Ala271–Thr288, observed experimentally in the solution structure of frog Nbs1 BRCT2^30^ (Figure S2). The TopBP1 crystal structure contains a similarly positioned but shorter disordered sequence (residues Asn1442-Ser1449)^31^. Although the hsNbs1 BRCT2 domain was modeled using the TopBP1 BRCT8 template, the secondary structure elements align closely with those determined by NMR for *X. laevis* Nbs1 BRCT2 domain (Figure S3)^30^. Atypically, TopBP1 BRCT7/8 exhibits high affinity for a phosphothreonine-containing peptide in the FANCJ helicase^31^ with sequence: SIYFpTPELYD. Important characteristics of TopBP1 phosphopeptide recognition include lack of a strong pSer/pThr selectivity by the primary phosphopeptide recognition site, specific interactions favoring an anionic residue (Glu) in the + 2 position, and selectivity for hydrophobic residues at the + 3 and + 4 positions that interact with the BRCT7-BRCT8 interface. The lack of a strong pSer/pThr preference exhibited by the TopBP1 BRCT7/8 domains was attributed to an unusual peptide backbone position and pThr phosphate orientation resulting from interactions with Arg1280^31^. In the modeled hsNbs1 structure Lys125 replaces TopBP1 Arg1280, and its ε-amino group is 4 Å from the nearest phosphate oxygen, suggesting that hsNbs1 BRCT1 is likely to exhibit a more typical pSer/pThr preference.

The modeled BRCT1/2 domain appears to retain most of the features that would lead to similar peptide binding selectivity. TopBP1 Lys1317 aligns with hsNbs1 Lys160 which has previously been implicated in binding MDC1^3,17^, while TopBP1 Arg1280 corresponds to Lys125 in Nbs1 (Fig. 2B). In the TopBP1-FANCJ complex, the FANCJ Glu residue at the + 2 position extends into a depression on the surface of the domain, interacting with Gln1366 and Arg1314. In the modeled hsNbs1 structure, Gln1366 corresponds to Lys208, while Arg1314 corresponds to Val157. However, the adjacent Lys156 residue appears positioned to provide an additional electrostatic interaction with an anionic sidechain at the + 2 position. As outlined below, the retained selectivity of the + 2 site for an anionic residue provides an attractive explanation for the interaction of phosphorylated MDC1 with the BRCT1/2 domains.

Both TopBP1 and the modeled hsNbs1 BRCT1/2 domains include an inter-domain hydrophobic specificity pocket for the + 3/ + 4 positions that is formed from three helices: BRCT7 helix α2 and BRCT8 helices α5 and α8 (BRCT1/2 helices α2, α5 and α8) (Fig. 2C). Important interdomain binding pocket residues include: BRCT1: Val157, Ile159, Ile162; BRCT2: Lys237, Leu238, Ala241, Glu307, Ile310 (Figure S2B). Although these residues are identified using the TopBP1 BRCT7/8 model, all of the corresponding BRCT2 residues in the experimental xlBRCT2 structure are conserved and located in similar positions relative to the binding pocket (Figure S2). However, TopBP1 Arg1314 on helix α2 stacks against the Tyr ring of FANCJ, helping to select for an aromatic residue at + 4, while in hsNbs1 this residue corresponds to Val157. Hence, the BRCT1/2 binding pocket may be less likely to prefer an aromatic residue at + 4.

**Figure 3:**
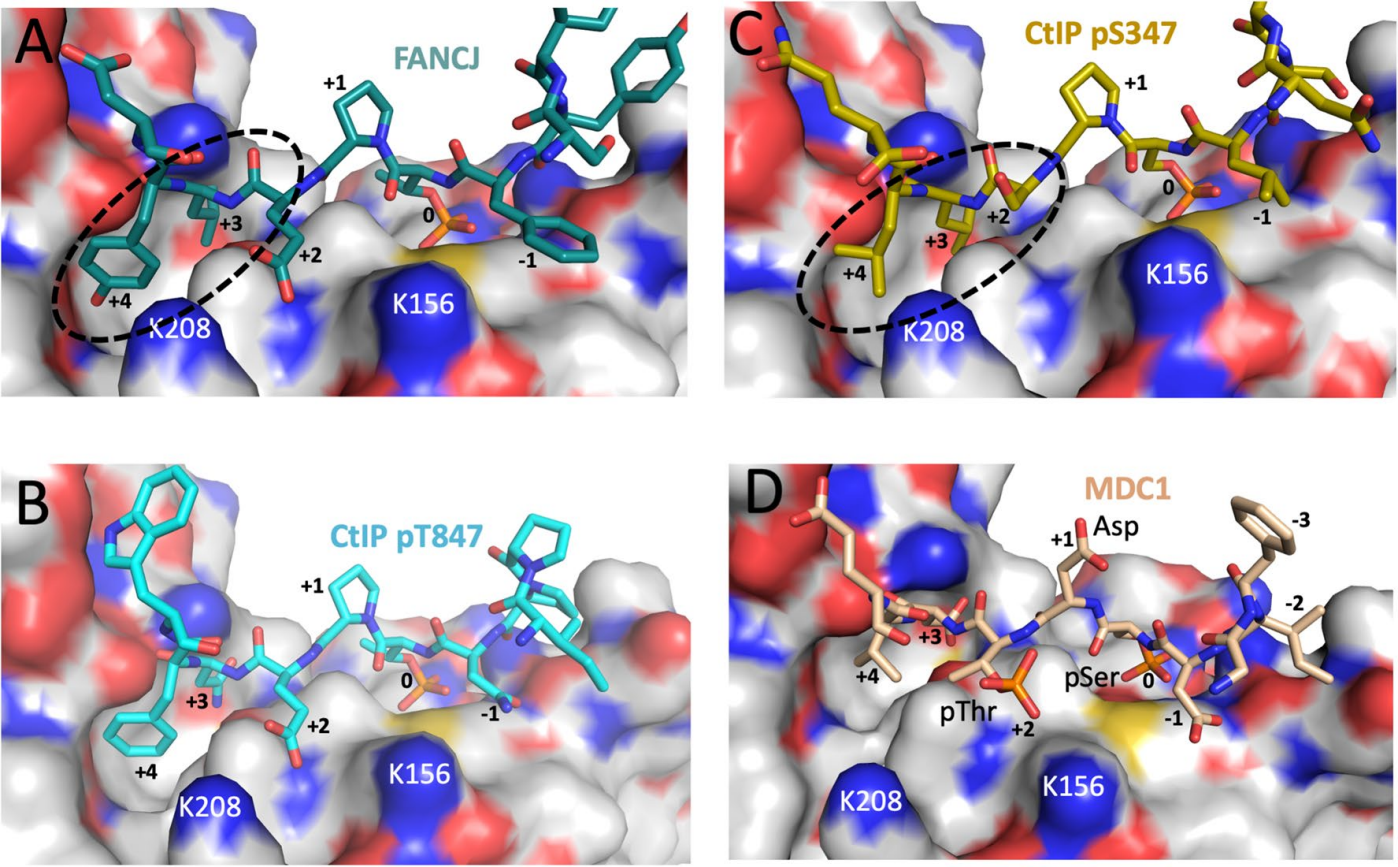
Interaction of modeled Nbs1 BRCT1/2 domains with phosphopeptide ligands. Surface rendering of modeled Nbs1 BRCT1/2 domains with: (**A**) the FANCJ phosphopeptide positioned as in Fig. 2; (**B**) CtIP pThr847 peptide; (**C**) CtIP pSer347 peptide; (**D**) MDC1 pSer-Asp-pThr peptide. Peptides were generated by mutating FANCJ without further optimization. The phosphopeptide binding pocket in BRCT1 is occupied by the position 0 pThr in panels (**A**) and (**B**), and by the position 0 pSer in panels (**C**) and (**D**). The peptide orientation illustrates the domain interactions with the anionic + 2 residue, and the BRCT1-BRCT2 interface hydrophobic binding pocket, also denoted by a dashed oval in panel C, for the + 3 and + 4 residues.

Overall, most of the features that confer binding selectivity are largely retained in hsNbs1 BRCT1/2. A surface rendering of the modeled hsNbs1 BRCT1/2 illustrates how interactions with various phosphopeptides discussed below are also consistent with the modeled structure (Fig. 3). The positively charged Lys156 and Lys208 residues probably play an important role in selectivity for an anionic residue at the + 2 position, which contributes to binding of Glu in the CtIP pThr847 peptide (Fig. 3B) and for binding pThr in the MDC1 pSer-Asp-pThr motifs (Fig. 3D). The relative positions of the FHA and BRCT1/2 domain phosphopeptide binding sites are illustrated in Figure S4.

### Interaction with a CtIP pThr847 peptide

**Figure 4:**
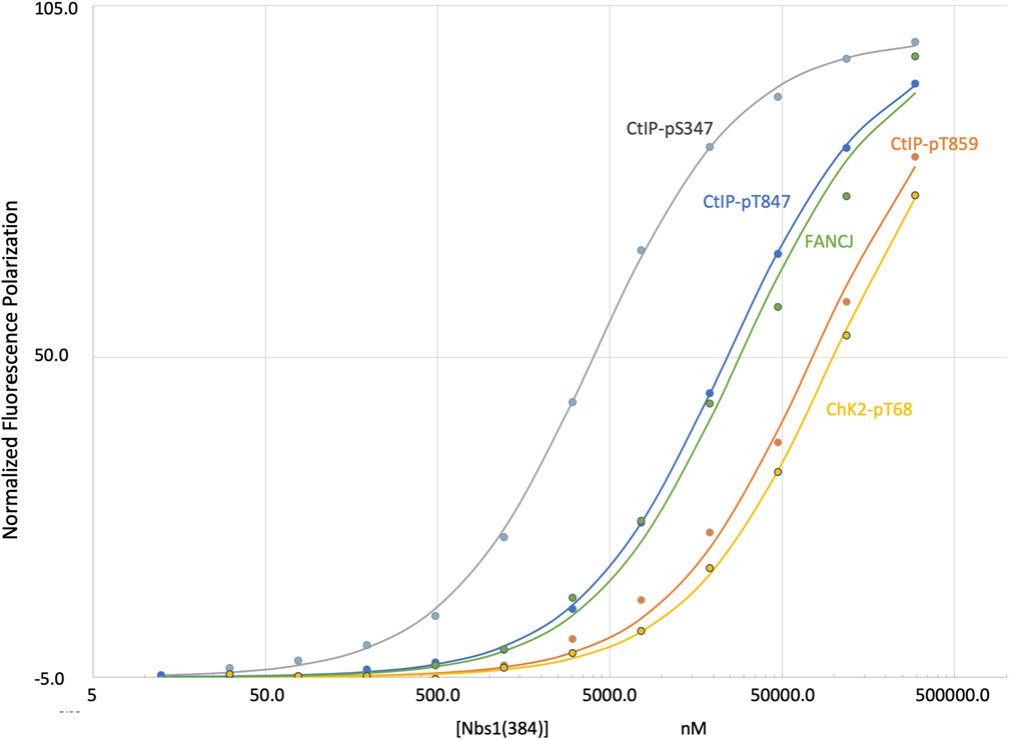
Binding of FITC-labeled peptides to hsNbs1(384). Solid lines with filled data markers show normalized fluorescence polarization of the following peptides (see Table 1 for peptide sequences) as a function of the concentration of hsNbs1(384) corresponding to: CtIP-pS347 (gray); CtIP-pT847 (blue); FANCJ (green); CtIP-859 (orange); ChK2-pT68 (yellow). FP studies were performed at room temperature in 25 mM HEPES (pH 7.4), 150 mM NaCl, 1 mM EDTA, 2 mM DTT, 0.05% Tween20, 0.1% BSA after a 15-min incubation period. The data points were fit to a single-site non-cooperative model.

The validity of TopBP1 BRCT7/8 as a model for Nbs1 BRCT1/2 was experimentally evaluated by measuring the affinity of the FANCJ phosphopeptide for Nbs1(384). A fluorescence polarization (FP) assay of a FANCJ pThr1133 phosphopeptide indicates moderate affinity binding for Nbs1(384) (K_d_ = 27 µM; Fig. 4, Table 1). The five FANCJ residues that directly interact most strongly with TopBP1: pTPELY, show significant homology with CtIP residues pT^847^PENF; CDK phosphorylation of Thr^847^ has been shown to support cell cycle control of DNA end resection^32^ and possibly binding to Nbs1^12,33^ (Fig. 3A,B). FP measurements of the Nbs1(384) dissociation constants for two CtIP pThr847 phosphopeptides gave values of 20 and 22 µM (Fig. 4, Tables 1 and 2). Based on the analysis presented by Leung et al.^31^ TopBP1 Arg1280 supports high affinity binding for pThr, so that the lower affinity of Nbs1 BRCT1/2 for both the FANCJ and CtIP pThr-containing peptides is likely to result at least in part from the replacement of Arg1280 with a Lys residue (Fig. 2B). Nbs1 is apparently able to accommodate either Asn-Phe or Leu-Tyr at the + 3/ + 4 position, but a + 4 hydrophobic residue appears to be essential. Consistent with these conclusions, another peptide sequence found to bind to TopBP1 BRCT7/8: RLApSNLQWPS contains Gln-Trp at the + 3/ + 4 positions^34^. Alternatively, the CtIP pSer327 peptide that targets the tandem BRCA1 BRCT domains^35,36^ contains Phe-Gly at these positions, but does not bind to Nbs1(384) (Table 1).

**Table 1:** Nbs1(384) phosphopeptide dissociation constants.

Protein	Phosphorylated residue	Sequence	K_d_^d^ (µM)
MDC1	pS329,pT331	^a^FID(**pS**)D(**pT**)DAEEE	0.65 ± 0.05
**CtIP**	pS269,pT271	^a^VQEE(**pS**)E(**pT**)QGPMSP	12.0 ± 2.0
"	pT312,pT315	TEDSLRFSDS(**pT**)SK(**pT**)PPQEELP^b^	90.0 ± 6.0
"	pS327	^a^TRVS(**pS**)PVFGAT	ND
"	pS347	GLDLNTSL(**pS**)PSLLQPGKKK^b^	2.64 ± 0.16
"	pT847	^a^RYIPPN(**pT**)PENFWEVG	22.0 ± 1.4
"	pT859	WEVGFPS(**pT**)QTCMERGYI^b^	90.5 ± 5.0
FANCJ	pT1133	EDESIYF(**pT**)PELYDPEIE^b^	26.9 ± 1.8
Chk2	pT68	SLETVS(**pT**)QELYSIPED^b^	98.7 ± 4.1
XRCC1	pS518,pT519	^a^DPYAG(**pSpT**)DENTDSEEHQE	> 65 µM
"	pS485,pT488	^a^DNGAED(**pS**)GD(**pT**)EDELR	34.8 ± 4.9
"	pS485,pT488	EGVQSEGQDNGAED(**pS**)GD(**pT**)EDELR^b^	4.5 ± 0.9
"	pS447	^a^AGPS(**pS**)PQKP	ND

^a^N-terminal FITC-GG-; ^b^C-terminal K/FITC, ^c^ND—binding not detected; ^d^all reported values are the mean of at least three measurements.

**Table 2:** Phosphopeptide dissociation constants determined for Nbs1 mutants.

Phosphopeptide^a^	hsNbs1(384)	Kd (µM)
**I: hsCtIP**		
R^841^YIPPNpTPENFWEVGFPEEQ^860^		
	wt	20.0 ± 0.92
FHA residues mutated	R28A	35.9 ± 2.60
"	R43A	29.2 ± 1.37
"	K29A	26.0 ± 1.08
"	K73A	32.9 ± 2.59
BRCT1 residues mutated	K125A	39.6 ± 4.93
"	K160A	132.6 ± 40.3
"	S118G	149.2 ± 33.6
**II: hsMDC1:**		
FITC-GG-FID(**pS**)D(**pT**)DAEEE	wt	0.65 ± 0.05
"	R28S,K29S,R43A	1.47 ± 0.04
FITC-GG-PFGFID**(pS)**D**(pT)**DVEEER	R28S,K29S,R43A	1.60 ± 0.02

^a^K_d_ values were determined by fluorescence polarization on the indicated peptides modified to include an N-terminal FITC-Gly-Gly.

### Mutational support for phosphopeptide binding targets and binding selectivity

**Figure 5:**
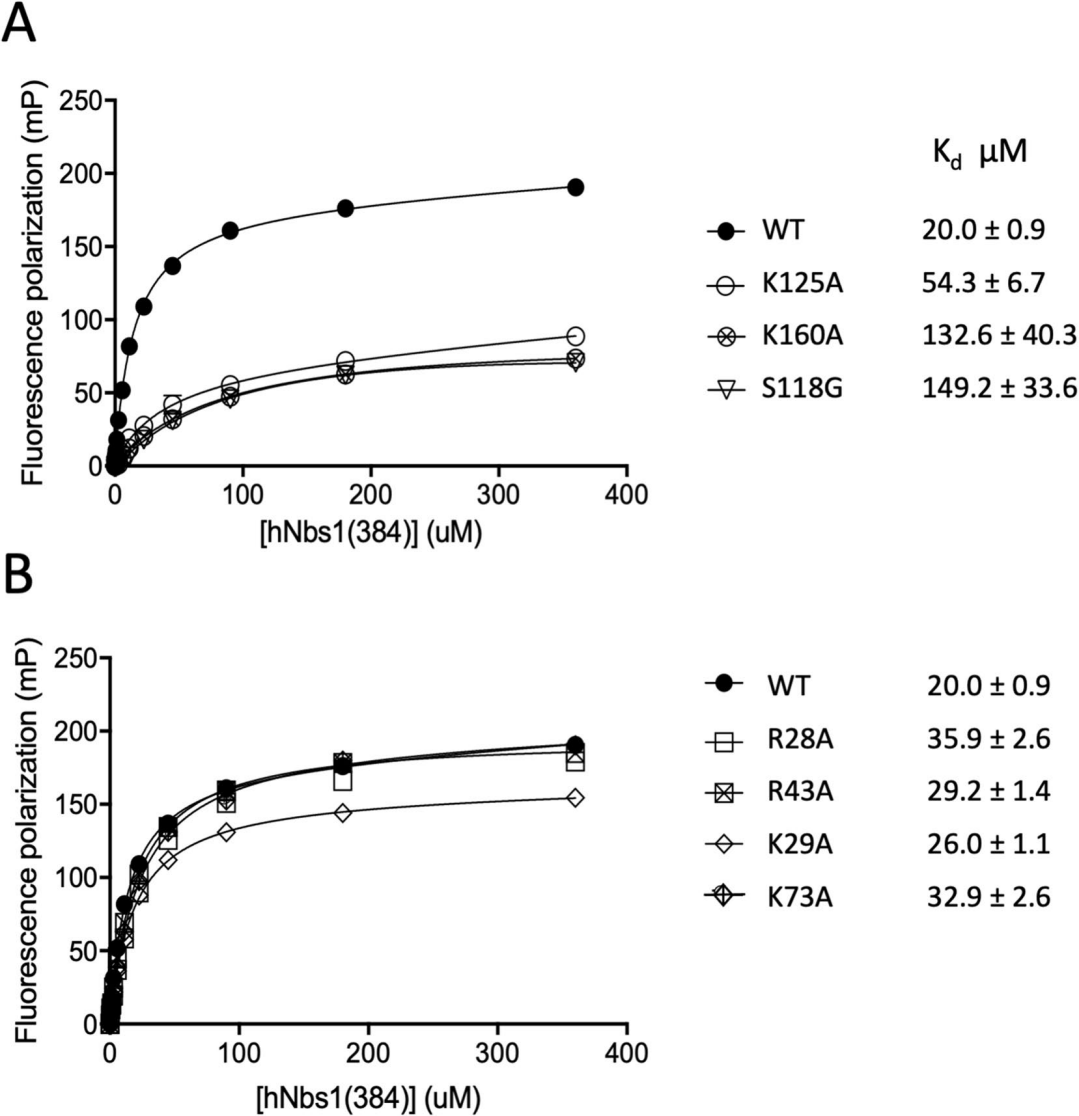
Effect of FHA and BRCT domain mutations on binding of CtIP pThr847 peptide. (**A**) Fluorescence polarization of a fluorescein-labeled CtIP pThr847 peptide (sequence given in Table 2) as a function of wild-type Nbs1 (384) concentration (filled circle) and for three BRCT domain variants: K125A (open circle), K160A (crossed circle), and S118G (inverted triangle). B) FP data for wild-type Nbs1(384) (filled circle) and four FHA domain variants: R28A (open square box), R43A (crossed square box), K29A (open diamond), and K73A (crossed diamond). Experimental conditions: FP studies were performed in 25 mM HEPES (pH 7.4), 150 mM NaCl, 1 mM EDTA, 2 mM DTT, 0.05% Tween20, 0.1% BSA after a 15-min incubation period.

In order to confirm the identity of the proposed FHA and BRCT binding sites and to evaluate how extensive any cross binding might be, we designed seven site-directed mutations: four that target the FHA binding site: R28A, K29A, R43A, and K73A, and three that target the tandem BRCT1/2 site: S118G, K125A, and K160A. The mutated residues were selected on the basis of models of the Nbs1 FHA-BRCT1/2 domains discussed above (Fig. 1). As shown in Fig. 5 and Table 2, the CtIP pThr847 peptide binds primarily to BRCT1, in contrast with the typical preference of the pThr-containing peptides for the FHA domain. Mutations of Ser118 and Lys160 have a larger effect on pThr847 peptide binding than the Lys125 mutation, indicating that the latter interaction, which differs from that in the TopBP1 BRCT7 domains is less significant for BRCT1 binding.

### Interaction of CDK-phosphorylated CtIP with the BRCT1/2 site

In a previous study, Wang et al.^7^ identified five CDK-phosphorylation sites located in the CtIP(200–400) segment that specifically support binding to both the Nbs1 N-terminal FHA and BRCT domains. Three phosphorylated Ser residues: pSer233, pSer276, and pSer347 were proposed to correspond to peptides targeting the BRCT1/2 domains, while the pThr245 and pThr315 were proposed to correspond to peptides targeting the FHA domain. Based on the analysis of BRCT1/2 summarized above indicating that the phosphopeptide binding site could also accommodate pSer and prefers hydrophobic residues at the + 3/ + 4 positions (Fig. 3C), the pSer347 peptide containing Leu residues at + 3 and + 4 was selected for further study; this peptide exhibits high affinity for hsNbs1, K_d_ = 2.64 ± 0.16 µM (Fig. 4, Table 1). Interestingly, the model places Ser349 in the + 2 binding pocket, suggesting that an even higher-affinity peptide ligand might be formed if Ser349 is additionally phosphorylated. We thus conclude that CDK-phosphorylated CtIP binding to the BRCT1/2 domains is likely to utilize pSer347.

### The bipartite binding of MDC1

MDC1 contains a segment, previously referred to as MDC1-SDT, corresponding to MDC1(141–621) by Hari et al.^17^, that is heavily phosphorylated and interacts with both the FHA and BRCT1/2 domains of Nbs1^3,17,21^. The Nbs1 FHA domain interacts directly with the pSer-Asp-pThr motif (Fig. 1), however the BRCT1 domain also interacts with the same or similar motifs in MDC1-SDT. In the modeled BRCT1/2 complex developed above, the pSer forms similar H-bond interactions with Ser118, Lys125, Lys160, and the Cys119 backbone bound to the principle BRCT1 phosphopeptide binding site. Additional peptide-protein interactions for the MDC1-BRCT1/2 complex are indicated by the molecular modeling studies described below.

Selective binding to the BRCT1/2 domains was demonstrated using an Nbs1 triple mutant: Nbs1(384)(R28S,K29S,R43A) in which three of the four FHA domain cationic residues thought to interact with the phosphopeptide were mutated. As shown in Table 2, both of two test peptides containing the pSer-Asp-pThr motif retain high affinity for the FHA domain-blocked variant. Further, the measured K_d_ value for the FHA domain-blocked mutant is about double that of Nbs1(384), consistent with the conclusion that this motif interacts with the FHA and BRCT1 sites with similar affinity. It thus appears that the observed binding to this variant arises from a specific interaction with the BRCT1/2 domains, and therefore that both FHA and BRCT1/2 binding interactions involve both phosphorylated residues of the pSer-Asp-pThr sequences.

### FHA domain recognition of phosphorylated CtIP

Although the CDK phosphorylation of CtIP Ser347 creates a peptide with micromolar affinity for the Nbs1 BRCT1/2 domains, the basis for high-affinity interaction with the FHA domain is less clear. Wang et al.^7^ identified two CtIP peptides containing SXT motifs: Ser-Tyr-Thr^245^ and Ser-Lys-Thr^315^ as supporting Nbs1 FHA domain binding. A NetPhos3.1^37^ analysis of CtIP indicates that both Thr245 and Thr315 are likely to be phosphorylated by CDK5, while Ser313 and, to a lesser extent, Ser243 are substrates for CDK1, making it less likely that these residues will be phosphorylated during the same part of the cell cycle. In addition, the Lys residue in the second peptide also might compete with FHA domain cationic residues for binding to pSer and pThr (Fig. 1). Wang et al. further identified phosphorylated peptides isolated from HeLa cells that were characterized by mass spectrometry (Table S1 of Wang et al.^7^). Consistent with the above analysis, peptides phosphorylated on Thr245 and Thr315 but not on Ser243 or Ser313 were identified. Some peptides containing pThr315 were additionally phosphorylated at Ser311 and/or Thr312. A test peptide containing the pThr^312^-Ser-Lys-pThr^315^ that includes two spacer residues shows very low affinity for Nbs1(384) (Table 1). We also analyzed CtIP sites more likely to undergo CK2 phosphorylation. A doubly phosphorylated CtIP pSer-Glu-pThr^271^ peptide also exhibits moderate binding affinity (K_d_ = 12 µM, Table 1).

### Molecular dynamics analysis of BRCT1/2-peptide complexes

We performed MD simulations on the modeled Nbs1 FHA and BRCT1/2 domains in the absence of ligands and in complexes with phosphopeptides corresponding to MDC1: GFID(pS)D(pT)DVE-NHCH_3_, CtIP pT847: IPPN(pT)PENFW-NHCH_3_, and CtIP pS347: NTSL(pS)PSLLQ-NHCH_3_. The initial positions of the peptides were derived using an overlay of the FANCJ-TopBP1 complex with the modeled Nbs1 BRCT1/2 domains, followed by mutating the FANCJ residues to the sequences in MDC1 and CtIP. The modeled FHA and BRCT1 domains exhibited stable, low RMSD values, while BRCT2 showed higher calculated RMSD and B-factor values (Figure S5). This behavior is qualitatively similar to that of the corresponding spNbs1 domains (Figure S6) and to the results of the crystal structure analysis^3^. Although a few regions of the FHA domain showed higher B-factors, these were not in the loops involved in phosphopeptide binding (Figs. 1, S5). Residues Ser118, Lys125 and Lys160 in the BRCT1 phosphopeptide binding site were extremely stable in the presence of MDC1, perhaps related to the specificity of the multiple H-bond and electrostatic interactions (Figure S7). Alternatively, Lys125 was more dynamic in the presence of the other two peptides, presumably alternating between various interactions with the phosphate group, the peptide backbone and perhaps other H-bond acceptors. Ser118 and Lys160 are conserved relative to the model TopBP1 structure, while Lys125 replaces Arg1280 in TopBP1 (Fig. 2B).

In order to gain further insight into the nature of peptide complexes, a small constraint (1 kcal/mol) was imposed on the backbone atoms, sufficient to maintain the general positions of the bound peptides. As indicated in Table 3, for calculations in which both the BRCT1/2 and peptide backbones are subject to the 1 kcal/mol backbone constraint, the two CtIP peptides exhibit favorable binding interactions with BRCT1, but show no significant interaction with BRCT2. The MDC1 peptide exhibits lower affinity for BRCT1, while there is a positive energy contribution (net repulsion) for the interaction with BRCT2. A second set of simulations constrained only the first five peptide backbone positions, leaving the last five residues unconstrained. Providing additional freedom for the last five peptide residues did not significantly alter the affinity of the two CtIP peptides for BRCT1, but enhanced their binding affinity to BRCT2 (Table 3). Interestingly, the binding affinity of the MDC1 peptide for BRCT1 became greater than that of the CtIP peptides, and the net repulsion for BRCT2 was greatly reduced. We performed multiple simulations using other constraint protocols, however none of these significantly altered the results or provided further insights.

**Table 3:** Calculated peptide-protein interaction free energies (kcal/mol).

Peptide	Peptide-BRCT1	Peptide-BRCT2
**I. Protein and peptide backbones constrained**		
MDC1	− 16.6 ± 5.4	16.2 ± 4.1
CtIP—pS347	− 25.5 ± 4.5	− 0.3 ± 2.3
CtIP—pT847	− 24.9 ± 6.9	3.8 ± 2.8
**II. The last five C-terminal residues of the peptide are not constrained**		
MDC1	− 36.1 ± 5.1	8.0 ± 3.6
CtIP—pS347	− 23.5 ± 4.4	− 12.9 ± 3.2
CtIP—pT847	− 26.5 ± 5.8	− 8.1 ± 3.5

**Figure 6:**
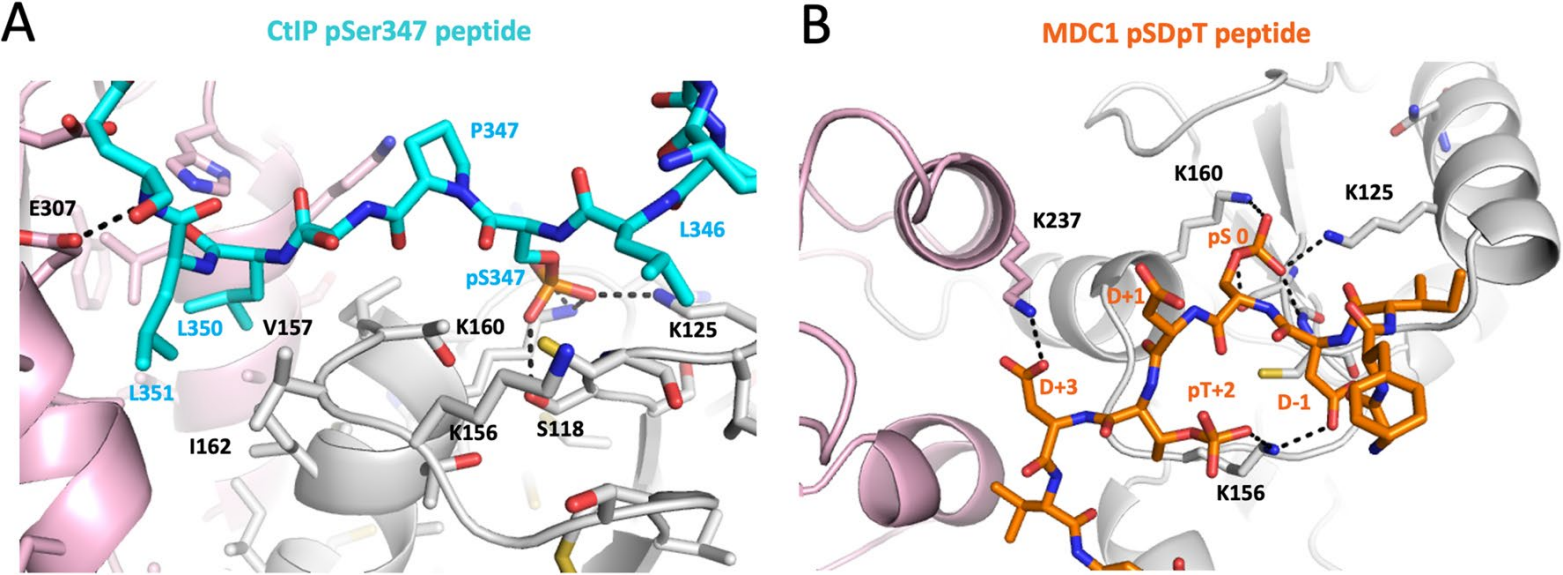
Modeled peptide-BRCT1/2 structures. Models were generated by constraining the position of the backbones of BRCT1/2 and of the first five peptide residues. (**A**) Optimized structure of the CtIP pS347 peptide-BRCT1/2 complex illustrating the interactions of Leu350-Leu351 with residues in the BRCT1-BRCT2 cleft. (**B**) Optimized structure of the MDC1-BRCT1/2 complex with pSer0 forming H-bonds with Ser118, Lys125, and Lys160 in the BRCT1 phosphate binding pocket. In the structure shown, Lys156 interacts with both pThr + 2 and with the Asp-1 sidechains. Lys237 interacts with the flanking Asp + 3 sidechain. Color coding: BRCT1 (gray), and BRCT2 (pink), pS347 peptide (cyan stick model), MDC1 peptide (orange stick model). H-bond lengths are given in Table S1.

Examination of typical calculated peptide-BRCT1/2 complexes provides a readily understood basis for the above observations (Fig. 6). Favorable interactions between the first phosphorylated residue with residues in the BRCT1 binding pocket: Lys125, Lys160, Ser118, and the Cys119 amide are generally maintained, although in some complexes Lys125 H-bonds to the peptide backbone. In addition, CtIP residues at the + 3 and + 4 positions for the pThr847 and pSer347 peptides (Fig. 6A) interact with the cleft between BRCT1 and BRCT2, consistent with the greater calculated binding affinity for the BRCT2 domain. TopBP1 binding pocket residue Arg1314 that stacks against the peptide + 4 Tyr residue is replaced by Val157 in the hsNbs1 BRCT1/2 model, eliminating the base stacking interaction. Val157 and other binding pocket residues that recognize the + 3/ + 4 residues also are well conserved in the frog BRCT2 structure (Figure S2B). The binding pockets of both hsTopBP1 BRCT7/8 and hsNbs1 BRCT1/2 are relatively narrow and deep, able to accommodate two consecutive sidechains and, for both proteins, at least two significantly different peptide sequences^31,34^. This contrasts with the more rigid BRCT repeats found in MDC1 and BRCA1 that accommodate only a single hydrophobic residue with high selectivity using a shallower binding pocket^38^.

For the MDC1 peptide, removing the position restraints for the last five peptide residues results in a significant improvement in the interaction with BRCT1 by allowing improved interaction of + 2 pThr331 with Lys156 and, in some simulations, Lys208, while pSer at position 0 remains in the primary phosphate binding pocket (Fig. 6B). Additional salt bridges involving Lys156 and Lys237 with the flanking Asp residues that precede and follow the pSDpT motif are also revealed in the modeled MDC1 complex (Fig. 6B). Lys156 may be more important for recognition of the + 2 residue than Lys208, since it is also conserved in fish and amphibian NBs1 (Figure S8). Based on the modeled hsNbs1 FHA and BRCT1/2 domain complexes, interactions with the Asp residues flanking the pSDpT sequence are involved in binding, suggesting that the CtIP DS^428^NT^430^D sequence may exhibit high affinity if doubly phosphorylated.

As discussed by Leung et al.^31^, the relative orientation of the two TopBP1 BRCT domains is significantly altered in response to peptide binding (Figure S9). Domain reorientation is accompanied by repositioning of Lys1317 (Lys160 in modeled Nbs1), linking this motion to changes in the BRCT1 phosphate binding site. The above simulations suggest that a similar change in the relative orientation of TopBP1 may result from interaction with the CtIP peptides, particularly in the case of the pSer347 peptide. Alternatively, the lack of a significant interaction of BRCT2 with the MDC1 peptide suggests that this interaction may not be accompanied by a similar conformational response. Interestingly, Williams et al.^9^ have observed that a peptide-FHA domain interaction in yeast Nbs1 that induces a 20° rotation of BRCT2 relative to BRCT1, further supporting the flexibility of the relative BRCT1-BRCT2 orientation and a possible peptide-dependent variation.

### Investigation of an XRCC1-Nbs1 phosphopeptide-dependent interaction

A direct interaction between phosphopeptide motifs in XRCC1 and Nbs1 has previously been reported^8^, and could be consistent with a role in recruitment of DNA Lig 3α binding partner of XRCC1, for participation in DSB repair via the alt-NHEJ pathway. XRCC1 phosphopeptides containing consecutively phosphorylated pSer^518^-pThr^519^ support binding to at least three FHA domains that are present in the proteins polynucleotide kinase/phosphatase (PNKP), aprataxin (APTX) and the NHEJ scaffold APLF^26–28^ and were evaluated for binding to the Nbs1 FHA domain. However, no interaction with Nbs1(384) was observed. We subsequently evaluated several other phosphorylated XRCC1-derived peptides and found that sequences containing pSer^485^-Gly-Asp-pThr^488^ show weak Nbs1 binding (Table 1). FP studies indicate that an XRCC1 peptide containing this sequence exhibits low affinity for Nbs1(384), and mutational studies indicate that this interaction involves the Nbs1 FHA domain. Residues Ser475, Ser485 and Thr488 are predicted to be phosphorylated by CK2 using the NetPhos 3.1 server^37^, and formation of these CK2-phosphorylated residues has been experimentally confirmed by Loizou et al.^39^. Peptides containing an N-terminal fluorescein tag showed modest affinity for Nbs(384), with K_d_ = 35 µM (Table 1), but surprisingly, a peptide containing a C-terminal fluorescein binds with much greater affinity, with K_d_ = 4.5 µM (Table 1). Fluorescein tags have occasionally been reported to influence binding affinity^40^, however this eightfold difference is the largest effect of this type that we have thus far encountered.

### Rodent XRCC1 contains an SDT motif

Although we were unable to identify a phosphorylated motif with high affinity for Nbs1 in human XRCC1, we unexpectedly found that linker 2 in most rodent XRCC1 includes SDT sequences located in polyanionic segments, indicating a high phosphorylation probability, as predicted by a NetPhos 3.1 analysis^37^ (Table 4). Based on the studies summarized in Table 3, these sequences should bind to both the FHA and BRCT1 domains. Although the cooperative effect resulting from multiple SDT sequences found in MDC1 will not be present, there still may be some cooperative binding involving the SDT sequence and one of the lower-affinity XRCC1 sequences such as the SGDT sequence described above that is also present in rodent XRCC1 about 20 residues downstream of the SDT sequence. In addition to affinity for Nbs1, phosphorylated SDT motifs have also been demonstrated to exhibit micromolar affinity for the FHA domains of APTX and PNKP^41^.

**Table 4:** Rodent XRCC1 SDT motifs.

Species	Sequence	pS prob^a^	pT prob^a^
Mus musculus—mouse	Q^464^DNSDTEGEESE	0.720	0.722
Mastomys coucha—Southern multimammate mouse	Q^464^DNSDTEGEQSE	0.674	0.657
Peromyscus maniculatus—deer mouse	Q^464^DNSDTEGEQSE	0.674	0.657
Chinchilla lanigera—long-tailed chinchilla	Q^465^ENSDTEEEESA	0.758	0.716
Cricetelus griseus—Chinese hamster	Q^466^DNSDTEGEQSE	0.658	0.657
Meriones unguiculatus—Mongolian gerbil	Q^449^DSSDTEGEQSE	0.671	0.642
Microtus ochrogaster—prairie vole	Q^488^DNSDTEGEQSE	0.674	0.657
Fukomys damarensis—Damara mole rat	Q^465^ENSDTEEEQSA	0.719	0.647
Heterocephalus glaber—naked mole rat	Q^460^ENSDTE-EQSA	0.697	0.503
Rattus norvegicus—Norway rat	Q^464^DNSDTDGEQSE	0.565	0.669
Octodon degus—common degu	Q^465^ENSDTDEEQSG	0.664	0.651

^a^CK2 phosphorylation probability evaluated using NetPhos 3.1^37^. Values > 0.5 are considered significant and correspond well with experimentally verified CK2 phosphorylation of XRCC1.

### Evaluation of other phosphopeptide binding interactions

The structural similarity between TopBP1 BRCT7/8 and Nbs1 BRCT1/2 identified by Swiss model led us to further evaluate whether other pThr-containing peptides with similar sequences and/or related functions might also be interacting with Nbs1. Wang et al.^7^ also reported that ATM-mediated phosphorylation of CtIP at Thr859 is essential for promoting end resection and homologous recombination. Checkpoint 2 kinase (Chk2) is a Ser/Thr protein kinase that senses DNA damage following phosphorylation by ATM and ATR1,2,3^42,43^. Initial phosphorylation of Thr68 creates a phosphopeptide with sequence: pTQELY, similar to the sequences of FANCJ pThr1133 and CtIP pThr847 peptides. However, neither peptide exhibits high-affinity Nbs1(384) binding (Table 1).

## Discussion

Phosphopeptide interactions involving the Nbs1 N-terminal FHA-BRCT1/2 domains play a central role in the DSB repair functions of the MRN complex^12,15,21^. However, cellular and biochemical studies of the behavior of Nbs1 as well as previous structural characterization of the *S. pombe* Nbs1 N-terminal domains have provided only limited insight into the remarkable ability of the FHA and BRCT domains to recognize motifs formed by both CK2- and CDK-dependent phosphorylation. In the present study, we found that the TopBP1 BRCT7/8 domains provide a reasonable template for modeling Nbs1 BRCT1/2, facilitating further analysis of its phosphopeptide binding partners. The validity of the model is supported by multiple observations, including the demonstration that the FANCJ peptide that binds to TopBP1 also binds to Nbs1 BRCT1/2, although with reduced affinity. Based on the modeled structure, the primary Nbs1 phosphopeptide recognition site in the tandem BRCT domains is located on BRCT1, which also favors the presence of an anionic residue at the + 2 site. Additional interactions involving the BRCT1/2 interface play a role in determining peptide specificity and may induce a conformational change.

Fluorescence polarization studies of fluorescein-labeled MDC1 peptides indicate that the same MDC1 pSer-Asp-Thr (pSDpT) motifs are recognized by *both* the FHA and BRCT1 domains. The pThr residue interacts with the primary phosphopeptide binding site with the -2 pSer residue binding to several cationic residues^3,9^ on the domain (Fig. 1). BRCT1 interacts with pSer at its major phosphopeptide recognition site, and with the +2 pThr at a  subsite that probably involves interactions with Lys156 and perhaps Lys208 (Figs. 3, 6). The modeled structure also identifies additional salt bridge interactions with Asp residues that flank the central pSDpT motif. These results are consistent with binding data reported by Hari et al.^17^ indicating that both the FHA and BRCT1 phosphopeptide binding sites support MDC1 binding, and with the mutational effects reported by Lloyd et al.^3^ The K_d_ value obtained using a fluorescein-labeled MDC1 peptide (Table 2) is similar to the value obtained by Lloyd et al. using isothermal titration calorimetry to evaluate the same peptide lacking a fluorescein label^3^. Substitution of a critical TopBP1 BRCT7 Arg1280 residue with the hsNbs1 BRCT1 Lys125 residue results in a more typical BRCT domain preference for pSer consistent with the pSer-Asp-pThr binding results in Table 2. The structure of the FHA domain-peptide complex (Fig. 1) reveals that the integrity of the binding site depends on the specific peptide bonds formed with pSDpT. Alternatively, the binding interactions of the BRCT1/2 domains are distributed on the peptide surface (Fig. 6), consistent with the lower specificity of this site which supports binding to two very different peptides—the CtIP pS347 and MDC1 pSDpT. This reduced degree of selectivity is also characteristic of TopBP1 BRCT7/8^31,34^.

In contrast with MDC1 binding, the interaction of hsNbs1 with CtIP relies on CDK-dependent phosphorylation motifs that have been mapped to a central region of CtIP^7^. Based on the modeled structure, we identified a CtIP pSer347 peptide from among several candidates identified by Wang et al.^7^ and verified micromolar affinity for Nbs1 (Fig. 3 and Table 1). However, we were less successful in identifying the basis for the reported bipartite interaction of CtIP with Nbs1 that also involves the FHA domain. Based on the above studies, CDK-dependent binding to the Nbs1 FHA domain is likely to depend primarily on the pSer-Lys-pThr^315^ motif that is phosphorylated on Ser and Thr by CDK1 and CDK5, respectively. However, none of the HeLa cell peptides isolated by Wang et al. was phosphorylated at Ser313, and the corresponding kinases are active during different parts of the cell cycle. Since CK2 is constitutively expressed, some additional FHA domain binding may involve CK2-phosphorylated CtIP motifs such as pSer-Glu-pThr^271^ (K_d_ = 12 µM, Table 1) or Asp-pSer^428^-Asn-pThr^430^-Asp, which contains flanking Asp residues. Interestingly, yeast Ctp1, the ortholog of human CtIP, was found to interact with the Nbs1 FHA domain via CK2 phosphorylation-dependent pSXpT motifs^9^. The above observations are consistent with studies indicating that the Nbs1 FHA domain may not be essential for the regulation of MRN activity by CtIP phosphorylation^33^.

The modeling studies indicate flexibility of the relative BRCT1-BRCT2 orientation that can be influenced by peptide binding (Figure S9), which is consistent with observations by Williams et al.^9^. These studies suggest that interaction with the CtIP pSer347 peptide may induce an orientation change, while MDC1 would not be expected to produce such a change since it fails to interact with the BRCT domain interface. A flexible FHA-BRCT1 interface previously observed to trap thiocyanate^9^ may also be able to interact with additional ligands.

The CDK-phosphorylated Thr847 residue is of critical importance for directing end resection of DSBs, fulfilling a role functionally analogous to that of pSer267 phosphorylation in *S. cerevisiae* Sae2^19,33^. However, it is unclear whether the K_d_ = 20 µM (Table 2) corresponds to a physiologically significant interaction. Further, the CtIP(T847E) mutant recovers much of the wild-type activity^32^, while the corresponding peptide would be expected to exhibit a significantly weaker interaction with Nbs1, with K_d_ > 20 µM. It is therefore unlikely that the pT847 peptide-Nbs1 BRCT1/2 interaction mediates the effects of CtIP on end resection.

An XRCC1 sequence containing pSer^485^-Gly-Asp-pThr^488^ that is related to the MDC1 pSer-Asp-pThr exhibits low affinity for Nbs1, similar to the lower-affinity binding observed for the interaction of MDC1 with TopBP1 BRCT4/5^44^, The additional Gly residue in the motif may lead to an intermolecular complex in which residues preceding or following Gly bind to different Nbs1 molecules, similar to the MDC1-TopBP1 BRCT4/5 interactions. Alternatively, linker 2 of rodent XRCC1 satisfies the three criteria for Nbs1 binding: an intrinsically disordered SDT-containing segment with high phosphorylation probability (Table 4). The reasons for a species-selective occurrence of a rodent XRCC1-Nbs1 interaction are unclear. However, the above studies reveal an interesting binding asymmetry: The phosphorylated SDT motif that targets the Nbs1 FHA domain also has been shown to bind with micromolar affinity to the FHA domains in the APTX and PNKP enzymes^41^. However, the XRCC1 Yxx(pS)(pT)D FHA-binding motif that interacts with the APTX and PNKP FHA domains lacks affinity for the Nbs1 FHA domain (Table 1).

## Materials and methods

### Protein preparation

The N-terminal 384 residues of homo sapiens Nbs1 (hsNbs1(384) containing the FHA-BRCT1-BRCT2 domains (NCBI reference sequence: NM_001024688) were optimized for expression in *E. coli* and cloned into pET30a (NdeI/HindIII) by GenScript. The construct was transformed into *E. coli* strain BL21(DE3) pLacI. Cells in one tenth of the total 2XYT media culture volume were grown overnight at 37 ºC and inoculated into the main culture. The main culture was incubated at 16 °C for 2 h for temperature equilibration prior to protein induction with 0.5 mM isopropyl β-D-1-thiogalactopyranoside. After induction, the culture was incubated for an additional 20 h at the same temperature. Harvested cells were disrupted by sonication and centrifuged at 40,000 × *g* for 20 m at 4 °C. The supernatant was applied to a Nickel-charged NTA column (prepacked 5 ml HisTrap NTA HP, GE healthcare) and eluted with imidazole. An ion exchange column (prepacked 5 ml HiTrap Q HP, GE healthcare) was employed for further purification. Finally, the highly-purified protein was obtained through a size exclusion column (Superdex 75 prep grade, GE healthcare) in 25 mM HEPES pH 7.5, 150 mM NaCl, 1 mM TCEP. Alternatively, hsNbs1(384) was expressed by autoinduction^45^ and purified by the above procedure. A shorter construct, hsNbs1(334) containing the three N-terminal domains was also expressed, but was less stable than the longer construct, and thus no FP data are reported for this construct. All protein samples were concentrated and stored at − 80 °C until used. Protein concentration was determined spectrophotometrically using the extinction coefficient values at 280 nm e280nm([hsNbs1(334)]) = 26,930 M^−1^ cm^−1^; e280nm (hsNbs1(384)]). = 33,920 M^−1^ cm^−1^.

The QuikChange site-directed mutagenesis kit (Agilent) was used to create an expression vector for the seven single-site variants of hsNbs1(384), corresponding to R28A, R43A, K29A and K73A in the FHA domain, and to S118G, K125A, and K160A in the BRCT1 domain. The hsNbs1(384)(R28S,K29S,R43A) triple mutant was also generated by the QuikChange method. Expression and purification of the variant proteins was the same as for the wild-type.

### Fluorescence polarization assay

Phosphopeptide-protein interactions were measured by a fluorescence polarization (FP) assay. 100 nM FITC-labeled phosphopeptides were incubated at room temperature for 15 min with the indicated concentrations of hsNbs1(384) in FP buffer composed of 25 mM HEPES, 150 mM NaCl, 1 mM EDTA, 2 mM DTT, 0.05% Tween20, 0.1% BSA, pH 7.4 prior to the measurement of fluorescence polarization (POLARstar Omega microplate reader, BMG Labtech). All samples were prepared in a 96-well black flat-bottomed polypropylene microplate and each well contained 50 µL. Each well was recorded by 100 flashes with an excitation wavelength of 485 nm, and detection of emission at 520 nm. Data for the interaction of a phosphopeptide with hsNbs1(384) were analyzed using non-linear fitting with a one-site total binding model^46^. All K_d_ values were determined in triplicate, and reported as mean ± standard deviation. All phosphopeptides used were synthesized by GenScript.

### Molecular dynamics simulations

The three hsNbs1 N-terminal domains are somewhat arbitrarily defined as: FHA (1–112); BRCT1(113–219); BRCT2(220–329), based on structural comparisons with spNbs1 (PDB: 3HUF^9^, Xenopus laevis Nbs1 BRCT2 domain (PDB: 2K2W^30^), and hsTopBP1 BRCT7/8 (PDB: 3AL3^31^). Swiss model^29^ was used to generate the initial models for the hsNbs1 FHA domain using spNbs1 FHA domain as a template (PDB: 3HUF^9^), and for the hsNbs1 BRCT1/2 domains using TopBP1 BRCT7/8 domains as a template (PDB 3AL3). MD simulations were performed on both the modeled domains and on the spNbs1 (PDB: 3HUF) and TopBP1 BRCT7/8 (PDB: 3AL3) template structures for comparison. These systems were solvated in water boxes with the number of water molecules ranging from 15,000 to 24,000. The initial equilibration using the following protocol was implemented: All systems were subjected to (1) 500 ps belly dynamics with fixed protein heavy atoms, (2) minimization, (3) low temperature constant pressure dynamics at fixed protein to assure a reasonable starting density, (4) minimization, (5) step-wise heating MD at constant volume, and (6) constant volume simulation for 10 ns with a constraint force constant of 10 kcal/mol applied only on backbone heavy atoms. The next 50–100 ns was used to step-wise reduce the constraint force constant to zero. NPT molecular dynamics (MD) simulations were performed for these systems over 300 ns using the PMEMD module of Amber18^47^. All MD trajectories were calculated using the PMEMD module of Amber18 with 1 fs time step. The amino acid parameters were selected from the SB14ff force field of Amber18. Root mean squared deviations and B-factors of these systems were calculated using the analysis package CPPTRAJ in Amber18.

Interaction energies of hsNbs1 BRCT1 and BRCT2 domains were calculated using molecular dynamics (MD) simulations by the following procedure. Starting with the TopBP1 BRCT7/8 structures of unliganded (PDB: 3AL2) and peptide bound (PDB: 3AL3) complexes, initial hsNbs1 BRCT1/2 structures were created using Swiss model^29^. The bound peptide was appropriately mutated to obtain MDC1, CtIP pS347, and CtIP pT847 peptides structures. Using the tleap module of Amber18^47^ the complexes were solvated in a box of water with about 14,000 water molecules. Initial equilibration simulations were performed for about 50 ns with the protocol described above, followed by a 50 ns MD with the step-wise reduction of the force constant to 1.0 kcal/mol. Subsequent MD simulations were extended for 100 ns with 1.0 kcal/mol constraint force constant (applied only on backbone heavy atoms), then for another 100 ns with 0.5 kcal/mol force constant, and finally 100 ns of unconstrained simulations. Since the unconstrained simulations disrupted peptide binding, the final conformations of 1.0 kcal/mol constrained-force simulations (run prior to the unconstrained simulations) were selected as starting positions for another round of 100 ns simulations, after removing the backbone constraints (1 kcal/mol) only on the last five residues of the peptide. Using 100 configurations from each simulation selected at 1 ns interval, interaction free energies were estimated with the MMGBSA module of Amber18, at a salt concentration of 150 mM. For MMPBSA energy partitioning, the N-terminal 107 residue segment (Pro112-Lys219) was considered to be in BRCT1 while the rest was assigned to BRCT2.

## Supplementary Information

Supplementary Information.
