# Supplementary material for: Phosphopeptide interactions of the Nbs1 N-terminal FHA-BRCT1/2 domains
Source: Sci Rep. 2021 Apr 27;11:9046. doi: 10.1038/s41598-021-88400-7 (PMC8079451; doi:10.1038/s41598-021-88400-7)
Supplement: Supplementary file 1 — Supplementary Information. [file 41598_2021_88400_MOESM1_ESM.pdf]

Supplementary Tables and Figures

Phosphopeptide interactions of the Nbs1 N-terminal FHA/BRCT1/2 domains

By Kyungmin Kim, Thomas W. Kirby, Lalith Perera, and Robert E. London

Table S1. Intermolecular peptide-protein bond lengths corresponding to Figs. 1 & 6 structures

Panel A: spNbs1 FHA domain-Ctp1 peptide bond lengths[a]			
Ctp1 residue	atom	spNbs1 residue	Bond length (Å)
pSer77[a]	OG	R27-NZH	3.4
	O3P	R27-NEH	3.4
	"	N28-NDH	2.7
	CO	R27-NZH2	2.9
Thr78			
pThr79	CO	K76-NH_2	2.6
	OG1	R27-NH1	3.1
	O1P	S44-OG	3.2
	O2P	-	
	O3P	K45-NH	3.2
	"	K45-NZH	3.5
	"	R27-NH2	3.5
Asp+1	NH	K41-CO	3.5
	OD1	K41-NZH	3.7
Glu+2	OE2	K104-NZH	2.7
Panel B: modeled hsNbs1 FHA-peptide bond lengths[b]			
Peptide residue	atom	hsNbs1 residue	
Asp-3			
pSer-2	CO	R28-NH_2	2.8
	OG	R28-NH_2	3.5
	O1P	K29-NZH	3.1
	O2P	R43-NE	2.8
	O3P	R28-NEH	3.7
Asp-1	CO2	K73-NH_2	2.8
pThr 0	CO	K73-NH	2.8
	OG1	R28-NH_2	3.0
		R28-NH_2	3.3
	O1P	S42-OG	2.8
	O2P	K73-NZH	2.8

	O3P	R43-NH	3.1
Asp +1			
Glu +2	CO	Q39-NEH	2.8
	OE2	Y74-OH	2.8

[a]All bond lengths other than those involving the modeled pSer77 sidechain are taken from the structure by Williams et al.[1], PDB: 3HUF.
[b]Distances obtained from modeled peptide-hsNbs1 FHA domain complex.
Distances up to 3.7 Å have been included, however the H-bonding significance of the longer distances is for the reader to determine.

FHA(1-112)-BRCT1(113-219)-BRCT2(220-328)

1 MWKLLPAAGP AGGEPYRLLT GVEYVVG***RK***N CAILIEND***Q***S I***SR***NHAVLTA NFSVTNLSQT
61 DEIPVLTLKD NS***KY***GTFVNE EKMQNGFSRT LKSGDGITFG VFGSKFRIEY EPLVACS*SC*L
121 DVSG*K*TALNQ AILQLGGFTV NNWTEECTHL VMVSVKVTI*K* TICALICGRP IVKPEYFTEF
181 LKAVESKKQP PQIESFYPPL DEPSIGSKNV DLSGRQERKQ IFKGKTFIFL NAKQHKKLSS
241 AVVFGGGEAR LITEENEEEH NFFLAPGTCV VDTGITNSQT LIPDCQKKWI QSIMDMLQRQ
301 GLRPIPEAEI GLAVIFMTTK NYCDPQGHPS TGLK

Figure S1. The hsNbs1 N-terminal FHA-BRCT1-BRCT2 domains are highlighted in yellow, green, and blue, respectively. Although we initially worked with the 334 residue Nbs1 construct, we found that a longer construct similar to that used by Lloyd et al.[2] was more stable, and the longer construct was employed in all reported studies. Phosphothreonine-binding residues in the FHA domain are italicized and bold-faced. Phosphoserine/phosphothreonine-binding residues in BRCT1 are italicized and underlined. Residues in the specificity binding pocket located at the interface of the BRCT1-BRCT2 domains are outlined and identified below:

BRCT1: Val157, Ile159, Ile162; **BRCT2:** Lys237, Leu238, Ala241, Glu307, Ile310.

The human BRCT2 binding pocket residues also align with xlBRCT2 residues: Lys235, Leu236, Ala239, Glu304 and Ile307.

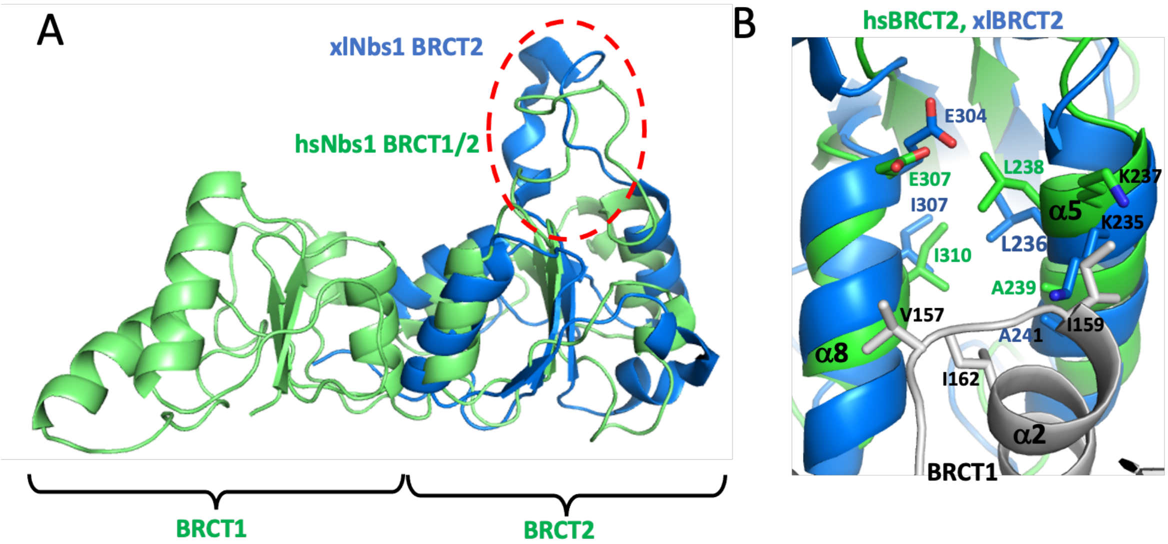

Figure S2. Comparison of modeled human Nbs1 BRCT1/2 domains with the solution structure of the Xenopus laevis Nbs1 BRCT2 domain. A) Ribbon diagram of modeled hsNbs1 BRCT1/2 domains (green), overlaid with xlNbs1 BRCT2 (PDB: 2K2W, [3], blue). The frog and human domains share 55.3% sequence identity and both contain a disordered largely hydrophobic segment corresponding to xlNbs1 residues Ala271-Thr288, which aligns with hsNbs1 residues Gly274-Gln291 (indicated by dashed red oval). In general, the more poorly aligned regions of BRCT2 are distal to the BRCT1-BRCT2 interface and exhibit lower sequence identity. B) Residues suggested by modeling studies to lie in the BRCT1-BRCT2 interdomain binding pocket that recognizes the phosphopeptide +3/+4 residues. Overlay of the modeled human and the experimental frog BRCT2 domain structures show that the locations of the BRCT2 residues are conserved.

Secondary structure identification of xlNbs1 BRCT2 and modeled hsNbs1 BRCT2

Sequence identity:
1: hsBRCT2 100.00 51.89
2: xlBRCT2 51.89 100.00

Clustal omega alignment of H. sapiens and X. laevis BRCT2 sequences

```
                   β1            α1      β2      α′           β3
hsBRCT2  ----IFKGKTFIFLNAKQHKKLSSAVVFGGGEARLITEENEEEHNFFLAPGTCVVDTGIT   56
xlBRCT2  KRKSIFKDKVFLFLNAKQYKKLSPAVLFGGGKTDLLMGELK-DASVLDNPATCVIDVAMT   59
             ***.*.*:******:**** **:****:: *:  * : : ..:  *.***:*..:*

             L3            α2        β4    α3              α"
hsBRCT2  NSQTLIPDCQKKWIQSIMDMLQRQGLRPIPEAEIGLAVIFMTTKNYCDPQGHPSTGLK     114
xlBRCT2  ESQLSESQSTQPWITSTLDLLQSKGLRTIPEAEIGLAVINVSTEIYCNPRR-------     110
         :**    :. : ** * :*:** :*** *********** ::*: **:*:
```

*Color code: b-strands, cyan, helices, magenta, disordered, yellow, highly distorted helix, gray.

Figure S3. Secondary structure identification of xlNbs1 BRCT2 and modeled hsNbs1 BRCT2. The frog and human sequences were aligned using Clustal omega.[4] The secondary structure of the hsNbs1 BRCT2 domain, modeled from the TopBRCT2 domain, is in very good agreement with the NMR-determined secondary structure of the Xenopus laevis Nbs1 BRCT2 domain.[3] In the human BRCT2, strand β2 is somewhat distorted but makes the standard H-bond interactions with the adjacent strand β1. PyMol identifies short helical segments α' and α" in the modeled BRCT2 domain, as well as a longer and more distorted helical segments in the frog BRCT that aligns with α' and with disordered loop L3. These are largely definitional issues and do not indicate significant secondary structure variations.

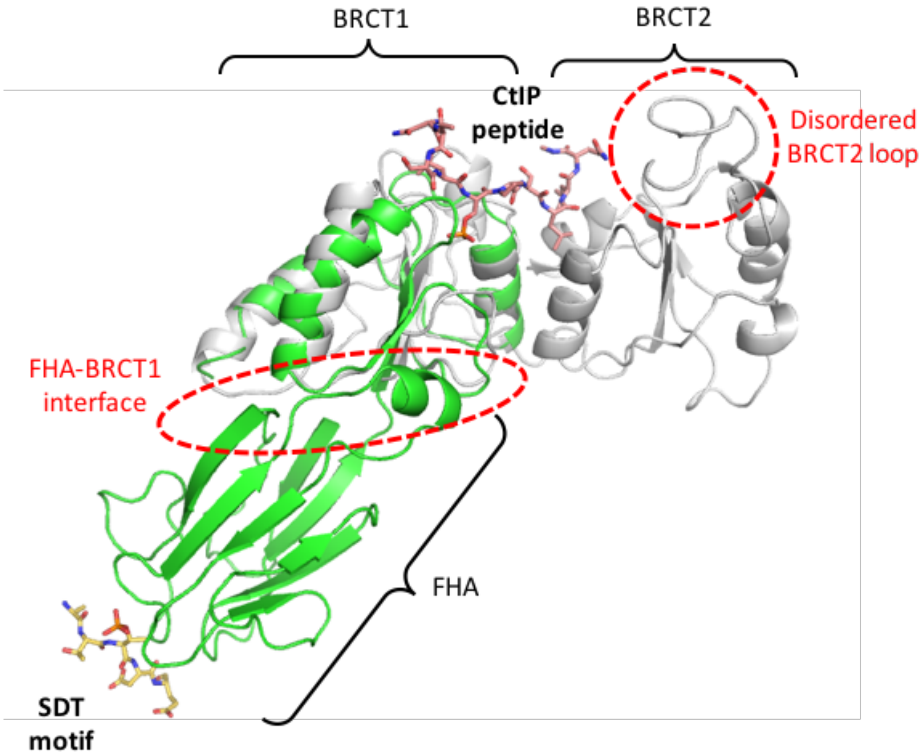

Figure S4. Overlay of modeled domains. The organization of the three human Nbs1 N-terminal domains can be obtained from an overlay of modeled hsBRCT1/2 domains (gray) with hsNbs1(1-186) (green) modeled using a spNbs1(1-190) template. This overlay relies on the homology of residues 110-190 of the spNbs1 with the first ~3/4 of the hsBRCT1 domain.[2] The overlay illustrates that the BRCT1 fragment modeled using spNbs1 or hsTopBP1 agree reasonably well (RMSD = 1.58). The differences between the human and yeast structures arise primarily in the loops and bent BRCT1 domain helix $\alpha 2$ (Val122-Leu135). Difficulties in modeling the isolated Nbs1 BRCT1-BRCT2 domains result from several factors including the absence of the FHA-BRCT1 interface and the disordered loop in the BRCT2 domain indicated by red dashed lines.

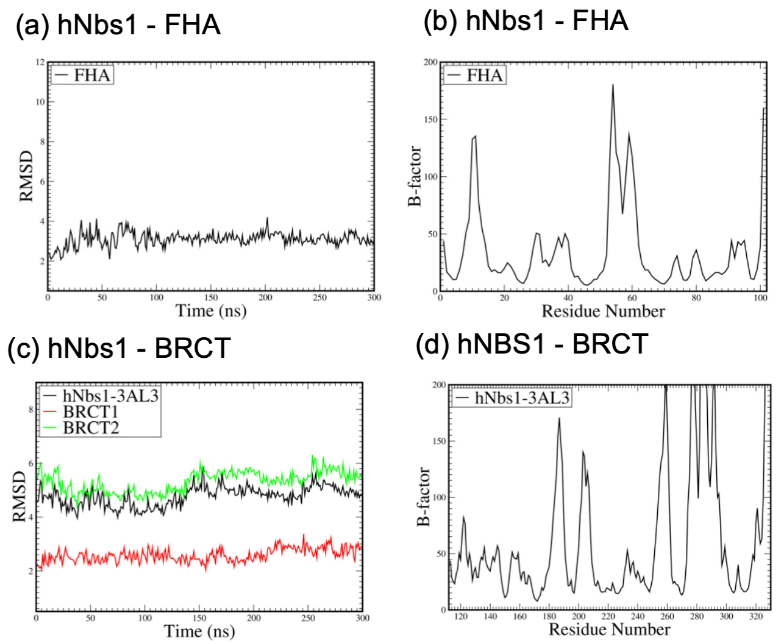

Figure S5. MD analysis of modeled hsNbs1 FHA and BRCT1/2 domains. Root mean square deviations (RMSD) of backbone heavy atoms as a function of time after an initial equilibration period for a) modeled hsNbs1 FHA, and c) modeled hsNbs1 BRCT1, BRCT2, and BRCT1/2 domains. Calculated B-factors for b) modeled hsNbs1 FHA based on the spNbs1 template structure (PDB: 3HUF[1]) and d) hsNbs1 BRCT1/2 modeled using TopBP1-BRCT7/8-FANCJ (pdb ID: 3AL3[5]). Further details for the MD methods and the templates used are provided in Methods. The modeled BRCT1/2 linker region is significantly more disordered if the apo TopBP1 (pdb ID: 3AL2[5]) structure is used as a modeling template (results not shown).

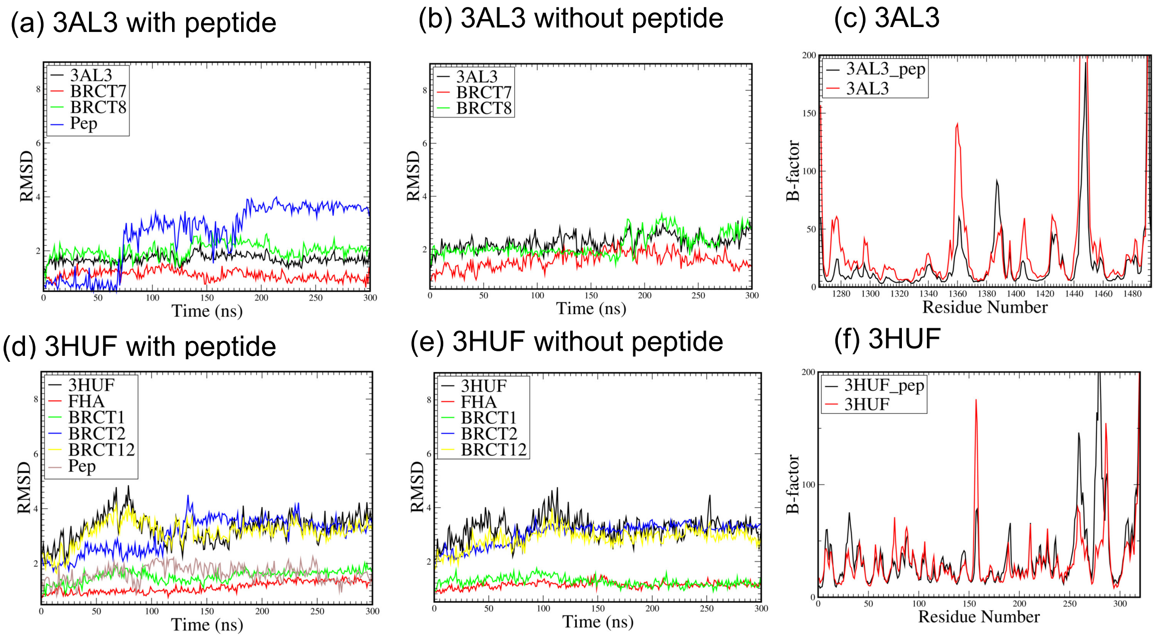

Figure S6. MD calculations for the spNbs1 FHA-BRCT1/2 and hsTopBP1 BRCT7/8 template structures. Heavy atom root mean squared deviation calculations for a) TopBP1-BRCT7/8-FANCJ (PDB: 3AL3[5]); b) TopBP1-BRCT7/8 without FANCJ but using PDB: 3AL3; d) spNbs1 complexed with Ctp1 (PDB: 3HUF); e) spNbs1 without the bound peptide (PDB: 3HUF[1]). B-factor calculations of c) TopBP1-BRCT7/8 (3AL3) including or excluding the peptide FANCJ; f) spNbs1 B-factor calculations with or without the bound peptide as indicated. The peptide FANCJ displayed larger RMSDs after 65 ns due to N-terminal repositioning within the BRCT domains. BRCT8 showed larger deviations both in RMSDs and B-factors.

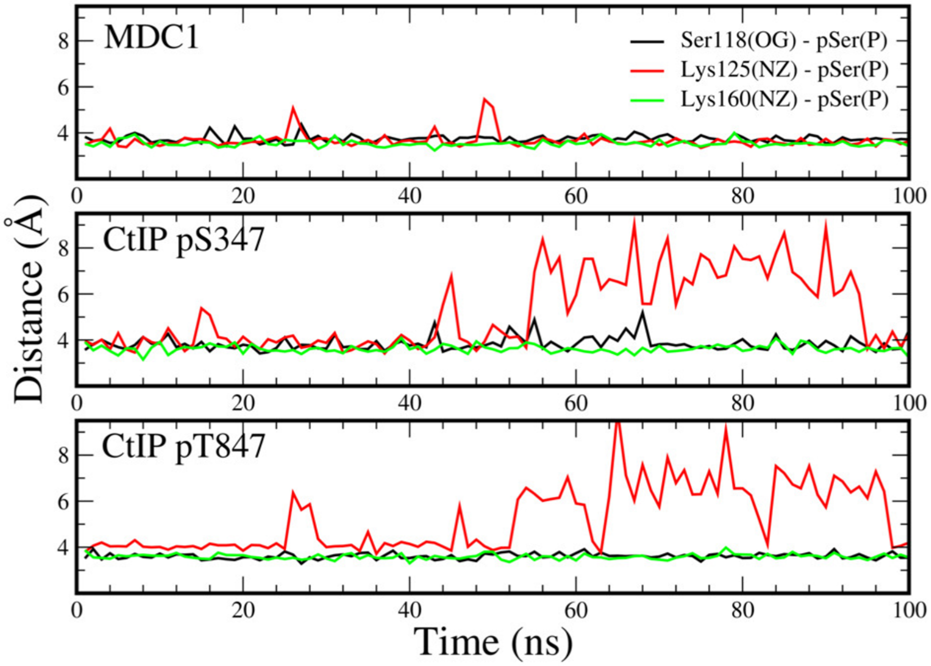

Figure S7. Stability of the BRCT1 phosphopeptide binding site. Plots of the time evolution of distances between the pSer or pThr phosphorus atom in the bound peptides and three nearby BRCT1 residues in the phosphopeptide binding pocket. Distances to Ser118 and Lys160 are well maintained, while the distance to Lys125 varies between phosphate-bound conformations and conformations consistent with alternate H-bonds.

Figure S8. Nbs1 Residue identity and alignments[a]

1: drNbs1	100.00	48.88	51.86	54.06	53.44	53.75	51.56	50.31
2: xlNbs1	48.88	100.00	56.52	57.72	56.17	57.10	55.42	55.11
3: ggNbs1	51.86	56.52	100.00	67.58	64.55	65.15	65.56	62.31
4: mmNbs1	54.06	57.72	67.58	100.00	83.53	82.63	85.29	82.18
5: hsNbs1	53.44	56.17	64.55	83.53	100.00	96.71	88.59	83.99
6: MaMuNbs1	53.75	57.10	65.15	82.63	96.71	100.00	88.89	83.99
7: ecNbs1	51.56	55.42	65.56	85.29	88.59	88.89	100.00	90.03
8: ooNbs1	50.31	55.11	62.31	82.18	83.99	83.99	90.03	100.00

```
drNbs1          -----------------------MWKLQPTE--SGGESVILLAGQEYVVGRKNCEILLTN  35
xlNbs1          -----------------------MWRLVAES--AAGGTYHFLTGTDYVVGRKNCAILIPE  35
ggNbs1          -----------------------MWKLVPAAG--PGEPFRLLVGTEYVVGRKNCAFLIQD  35
mmNbs1          -----------------------MWKLLPAAGAAPGEPYRLLAGVEYVVGRKNCGILIEN  37
hsNbs1          -----------------------MWKLLPAAGPAGGEPYRLLTGVEYVVGRKNCAILIEN  37
MaMuNbs1        -----------------------MWKLLPVAGPAGGEPYRLLTGVEYVVGRKNCAILIEN  37
ecNbs1          -----------------------MWKLVPVAGP-AREPYRLLTGVEYIVGRKNCGILIED  36
ooNbs1          MATTGKHNFGFVSEPFTVLSSQLKKFFSAFLAA-ENEPYRLLAGVEYIVGRKNCGILIED  59
                                          :             :*.* :*:****** :*: :

drNbs1          DQSISRVHAVLTVTEQ-----------AVTLKDSSKYGTFVNGEKLESGSTKTLQTGYKI  84
xlNbs1          DQSISRCHATLSVSHPSANLGQTNAASVLSIKDSSKYGTTVNGDKMNPAVPRNLKSGDKV  95
ggNbs1          DQSISRSHAVLTVSRPETTHSQSVSVPVLTIKDTSKYGTFVNGSKL-SGASRSLQSGDRV  94
mmNbs1          DQSISRNHAVLTVNFPVTSLSQTDEIPTLTIKDNSKYGTFVNEEKMQTGLSCTLKTGDRV  97
hsNbs1          DQSISRNHAVLTANFSVTNLSQTDEIPVLTLKDNSKYGTFVNEEKMQNGFSRTLKSGDGI  97
MaMuNbs1        DQSISRNHAVLTANFSVTNLSQTDEIPVLTLKDNSKYGTSVNEEKMQNGFSRTLKSGDSI  97
ecNbs1          DQSISRNHAVLTANFSVTNLSQTDEIPTLTIKDNSKYGTFVNEEKMQNGLSLTLKTRDRI  96
ooNbs1          DQSISRNHAVLTANFSVTNLSQTDEIPILTIKDNSKYGTFVNEEKMQNGLSRILKTGDRV  119
                ****** **.*:..              :::**.***** ** .*:  .    *::   :

drNbs1          TFGVFQSKFSLEKECIVVCSSCVDNEGKVTLSQDIRSVGGRLVSSWTSDCTHLVMPTVKV  144
xlNbs1          TFGVFNSKYRVEYEPLVVCSSCLDNSEKNSLNQNLIHLGGHVLNNWTEKSTHLVMTSIKV  155
ggNbs1          NFGVFESKFRVEYESLVVCSSCLDVAQKTALNEAIQQLGGLVVNEWTKECTHLIMESVKV  154
mmNbs1          TFGVFESKFRVEYEPLVVCSSCLDVSGKTVLNQAILQLGGLTANNWTEECTHLVMSAVKV  157
hsNbs1          TFGVFGSKFRIEYEPLVACSSCLDVSGKTALNQAILQLGGFTVNNWTEECTHLVMVSVKV  157
MaMuNbs1        TFGVFESKFRVEYEPLVACSSCLDVSGKTALNQAILQLGGFTVNNWTEECTHLVMVSVKV  157
ecNbs1          TFGVFESKFRVEYEPLVACSSCLDVAGKTALNQAVLQLGGLTVNNWTEECTHLVMISVKV  156
ooNbs1          TFGVFESKFRVEYEPLVACSSCLDVSGKTALSQAVLQLGGLTVNNWTEECTHLVMTSVKV  179
                .**** **: :* * :*.****:*   *  *.: :  :**   ..**...***:* ::**

drNbs1          TIKTICALLCCRPIVKPAFFSAFSKAVQQKLPLPKAERFRPQIDEPSLARDEVDLGARPE  204
xlNbs1          TIKTICALICCKPIIKPDYFCELLHAIQEKRPLPDYRSFIPSVDEPSLTPESLDLSENVK  215
ggNbs1          TVKTICALICGRPIVKPEFFSELMKAVQSRQQLPTPESFYPSVDEPAIGIDNMDLSGHPE  214
mmNbs1          TIKTICALICGRPIIKPEYFSEFLKAVESKKQPPDIESFYPPIDEPAIGSKSVDLSGRHE  217
hsNbs1          TIKTICALICGRPIVKPEYFTEFLKAVESKKQPPQIESFYPPLDEPSIGSKNVDLSGRQE  217
MaMuNbs1        TIKTICALICGRPIVKPEYFTEFLKAVQSKKQPPQIESFYPPLDEPSIGSKNVDLSGRQE  217
ecNbs1          TIKTICALICGRPIVKPEYFTEFLKAVQSKKQPPQIESFYPPIDEPAIGSKNIDLSGRQE  216
ooNbs1          TIKTICALICGRPIVKPEYFTEFLKTVQSKKQPPEIESFYPPIDEPVIGSKNIDLSERQE  239
                *:******:* :**:** :*  : ::::.:   *  . * * :*** :  ..:**. . :

drNbs1          RKSLFKGKTFLFLSSKQMKRLSVAVSCGGGVSQLLDEGA-LPVSLLESSSTCVLDMISGN  263
xlNbs1          RKSIFKDKVFLFLNAKQYKKLSPAVLFGGGKTDLLMGEL-KDASVLDNPATCVIDVAMTE  274
ggNbs1          RKKIFSGKTFVFLTAKQHKKLGPAVILGGGEAKLMAEER-KETSLLVSPEVCVVDVGVTN  273
mmNbs1          RKQIFKGKTFVFLNAKQHKKLSSAVAFGGGEARLMAEDDEEEQSFFSAPGTCVVDVGITN  277
hsNbs1          RKQIFKGKTFIFLNAKQHKKLSSAVVFGGGEARLITEENEEEHNFFLAPGTCVVDTGITN  277
MaMuNbs1        RKQIFKGKTFIFLSAKQHKKLSSAVVFGGGEARLITEENEEEHNFFLAPETCVVDIGITN  277
ecNbs1          RKQIFKGKTFVFLNAKQHKKLSSAVVFGGGDARLITEKNEEEDSFFSAPGTCVVDVGITD  276
ooNbs1          RKQIFKGKTFVFLNAKQHKKLSAAVVFGGGDARLITEDN-EEDSFFSAPGTCVVDVGITD  298
                **.:*..*.*:**.:** *:*. **  *** : *:        ..:    .**:*    :
```

```
drNbs1          SQPVISPASKKWLDSVGQILHRKGLRFITESEVGLAAIHVSNQTYCNPCSSLQSESVKTN  323
xlNbs1          SQLSESQSTQPWITSTLDLLQSKGLRTIPEAEIGLAVINVSTEIYCNPRR----------  324
ggNbs1          SQILGSESMRNWTDSILAVLESNNLRAIPEAEIGLAVIFMSTEIYCNPQRQPDNKAVTAS  333
mmNbs1          TQLIISHSQKKWIHLIMDTLQRNGLRPIPEAEIGLAVIFMTTENYCNPQGQPCTELK---  334
hsNbs1          SQTLIPDCQKKWIQSIMDMLQRQGLRPIPEAEIGLAVIFMTTKNYCDPQGHPSTGLK---  334
MaMuNbs1        SQTLIPDSQKKWIQSIMDMLQRQGLRPIPEAEIGLAVIFMTTKNYCDPRGHPSTGLK---  334
ecNbs1          SQNLIPDSQKKWIHSIMDMLQRQGLRPIPEAELGLAVIFMTTENYCDPQGQPSTGLKT--  334
ooNbs1          SQTLIPDSQKKRIHSIMDILQRQGLRPIPEAEIGLAVIFITTENYCDPQGQPSTGN----  354
                :*     . :         *. :.** * *:*:***.* ::.: **:*
```

[a]species abbreviations: hs, homo sapiens, ec, Equus caballus; MaMu, Macaque Mulatta; oo, Orcinus Orca, mm, mus musculus; gg, gallus gallus; xl, xenopus laevis, dr, danio rerio.
Important phosphate-binding residues and interdomain pocket residues are highlighted in yellow. Non-conserved residues are highlighted in cyan.

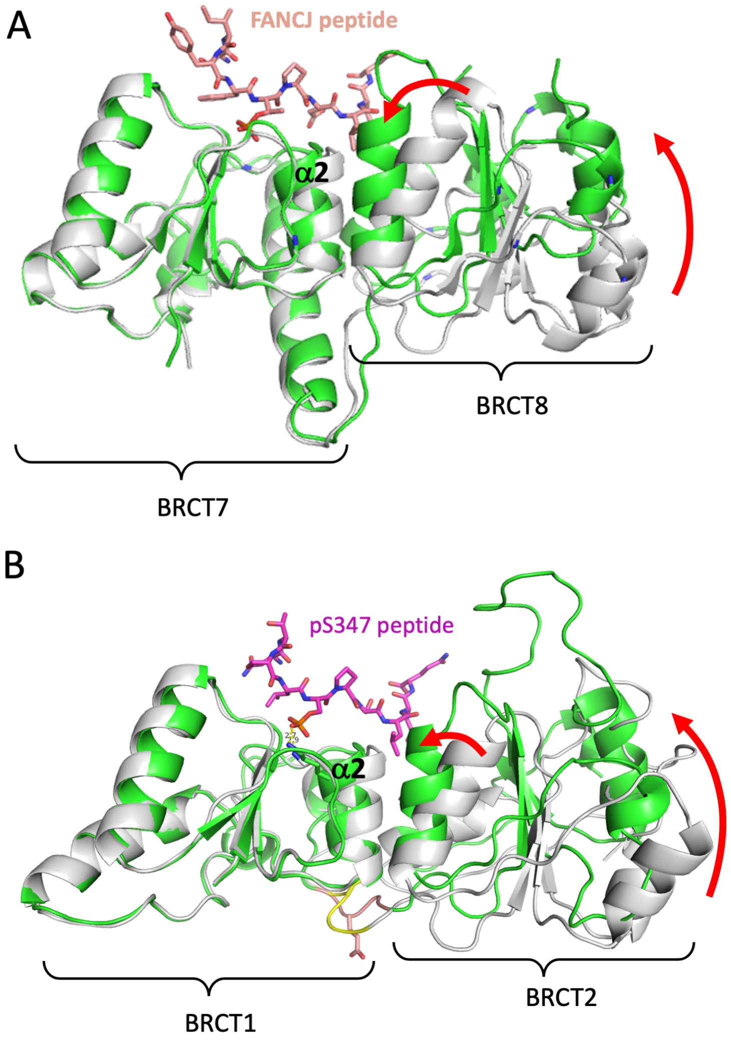

Figure S9. Conformational adjustments of the Nbs1 BRCT1-BRCT2 domains. A) Superimposed ribbon diagrams of the apo TopBP1 BRCT7/8 domains (gray, PDB: 3AL2) with the TopBP1-FANCJ peptide complex (green/beige stick figure; PDB: 3AL3 [5]). The BRCT7 domains are aligned to reveal the relative reorientation of BRCT8. B) Ribbon diagrams of the BRCT1/2 domains modeled using uncomplexed TopBP1 structure (gray, PDB: 3AL2) or the FANCJ peptide-complexed TopBP1 structure (green, PDB: 3AL3). In both structures, helix α2 in the first BRCT domain which is located at the BRCT domain interface tends to be pulled along with the second BRCT domain. The modeled Nbs1 BRCT1/2 domains are consistent with a possible peptide-induced conformational change of Nbs1 BRCT1/2, analogous to that of TopBP1.

References:

1 Williams, R. S. *et al.* Nbs1 flexibly tethers Ctp1 and Mre11-Rad50 to coordinate DNA double-strand break processing and repair. *Cell* **139**, 87-99, doi:10.1016/j.cell.2009.07.033 (2009).

2 Lloyd, J. *et al.* A supramodular FHA/BRCT-repeat architecture mediates Nbs1 adaptor function in response to DNA damage. *Cell* **139**, 100-111, doi:10.1016/j.cell.2009.07.043 (2009).

3 Xu, C. *et al.* Structure of a second BRCT domain identified in the nijmegen breakage syndrome protein Nbs1 and its function in an MDC1-dependent localization of Nbs1 to DNA damage sites. *J Mol Biol* **381**, 361-372, doi:10.1016/j.jmb.2008.05.087 (2008).

4 Sievers, F. & Higgins, D. G. Clustal Omega, accurate alignment of very large numbers of sequences. *Methods Mol Biol* **1079**, 105-116, doi:10.1007/978-1-62703-646-7_6 (2014).

5 Leung, C. C., Gong, Z., Chen, J. & Glover, J. N. Molecular basis of BACH1/FANCJ recognition by TopBP1 in DNA replication checkpoint control. *J Biol Chem* **286**, 4292-4301, doi:10.1074/jbc.M110.189555 (2011).
